# Stress and displacement patterns during orthodontic intervention in the maxilla of patients with cleft palate analyzed by finite element analysis: a systematic review

**DOI:** 10.1186/s12903-023-02714-8

**Published:** 2023-02-13

**Authors:** Mikulewicz Marcin, Chojnacka Katarzyna

**Affiliations:** 1 Division of Facial Abnormalities, Department of Dentofacial Orthopaedics and Orthodontics, Medical University of Wroclaw, ul. Krakowska 26, 50-425 Wrocław, Poland; 2 Department of Advanced Material Technologies, Faculty of Chemistry, Wroclaw University of Technology, Wrocław, Poland

**Keywords:** Cleft palate, Finite element analysis, Orthodontic treatment

## Abstract

**Objective:**

Rationale for the review in the context of what is already known about the evaluation of stress and displacement patterns using finite element analysis in the maxilla of patients with cleft palate after orthodontic intervention.

**Methods:**

This systematic review followed the Preferred Reporting Items for Systematic Reviews and Meta-analyses (PRISMA). The protocol for this systematic review was registered with PROSPERO (CRD42020177494). The following databases were screened: Medline (via PubMed), Scopus, Embase, and Web of Science.

**Results:**

The search identified 31 records. 15 articles were retrieved for full texts and 11 of them were considered eligible for inclusion by 2 authors. Eventually, 11 articles were included in the qualitative analysis.

**Conclusions:**

Finite element analysis is an appropriate tool for studying and predicting force application points for better controlled expansion in patients with UCLP.

## Introduction

Cleft lip and palate (CLP) is known to be the most common among other congenital disorders in the craniofacial region, with an estimated incidence of approximately 1: 1000 live births [1]. Usually, the medical staff involved in the treatment process is organized into a cleft team consisting of many specialists. Orthodontic treatment in patients with cleft palate involves various types of therapies/applications, can begin nearly after birth with naso-alveolar moulding (NAM) and can be performed until the end of growth (potential orthognathic surgery procedures) [2, 3]. The potential possibility of accurate simulation of the movement of the clefted parts of the maxilla with the use of different orthodontic appliances might be an important point in treatment planning.

One of the methods that can predict the rehabilitation carried out is the finite element method (FEM) [4]. Finite element analysis (FEA) is a modern computer simulation method that has found application in orthodontics in recent years as a simulation method of the force applied to bone / teeth and prediction of its displacement [5, 6]. In FEA analysis, based on patient CT scans, virtual models of anatomical structures are created. The most significant is to evaluate the characteristics of tissues (cartilage, bone, soft tissues, teeth) [5]. In the FEA approach, a single 3D model undergoes simulation to point out *elements* of mechanics in orthodontic treatment, particular distribution of stress in TADS and archwires, distribution of strain and stress in biological tissues (bones, teeth, PDL), conditions of resorption of roots, directions and the size of displacement for positioning of orthodontic appliance [7]. In FEA an important issue concerns individual variations and response to the applied forces, e.g. different rate, amount and directions of canine distal movement.

The approach in FEA orthodontic studies includes mostly biomechanics of the skull, assessment of (bio)mechanical loads of the skull, and mechanism of tooth movement. There are different aspects of using FEA in dentistry, for example, data on displacement, tension during chewing, distribution of mechanical forces on craniofacial forces [8]. FEA makes it possible to model and reproduce the anatomy of the craniofacial skeleton that helps to understand the parameters that influence bone remodelling [9].

FEA aims to simulate the conditions under which analysis would not be possible by clinical means. However, there are some disadvantages of this technique: complexity of craniofacial structures and their relationship, different information of tissue characteristics, limitation of anatomic details in reproduced models.

Geometric methods of analysis have limitations due to the nonregular and complex structure and shape of human bone. 3-D FEA enables investigating, e.g. reconstruction of alveolar bone and modifications of soft tissue making it useful and the errors of measurements can be reduced. However, it is important to link the model, prototype, and clinical treatment implemented.

### Aim of the study

Reason for the review in the context of what is already known about the evaluation of stress and displacement patterns using finite element analysis in the maxilla of patients with cleft palate after orthodontic intervention.

## Materials and methods

### Protocol

This systematic review followed the Preferred Reporting Items for Systematic Reviews and Meta-analyses (PRISMA). The protocol for this systematic review was registered with PROSPERO (CRD42020177494).

### Eligibility criteria

The Population, Intervention, Comparison, Outcome and Study Design (PICOS) framework was followed. The population was defined as patients with cleft palate, intervention, and orthodontic treatment that includes fixed or removable appliances. The comparison, stress and displacement pattern evaluation in the maxilla using finite element analysis. The primary outcome—evaluation of the results of the orthodontic intervention performed using finite element analysis. The secondary outcome was the diagnostic precision of the prediction of the 3D finite element model in the clefted maxilla. The study design—the following exclusion criteria were: (1) papers describing patients with no clefts, (2) studies without orthodontic intervention (3) repetitive publications, (4) animal studies, (5) reviews.

### Search strategy and study selection

**Fig. 1:**
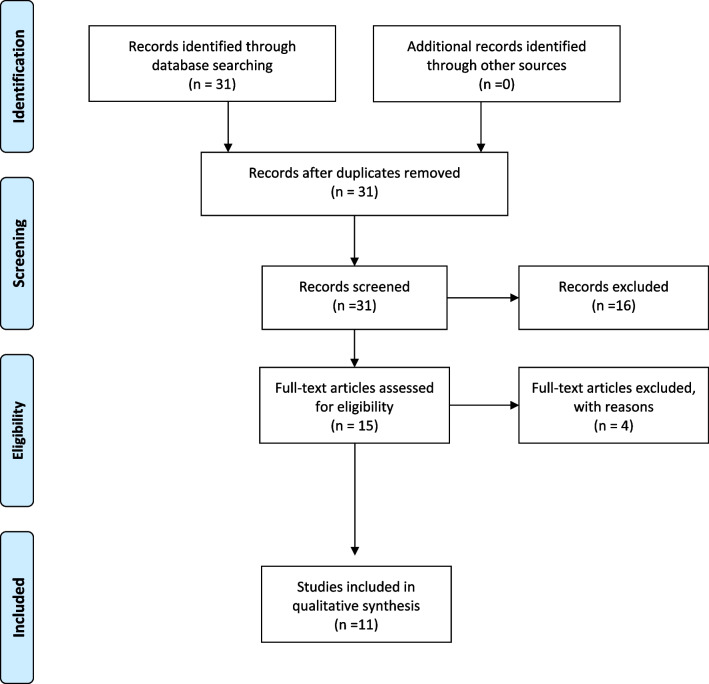
Prisma diagram (PRISMA flow chart)

The following databases were screened: Medline (via PubMed), Scopus, Embase and Web of Science (from 1970.01.01 to 2021.12.31) (Fig. 1). The search strategy combined MeSH heading words with free text words. The main search terms used were “finite element analysis and cleft palate and model (stress or displacement pattern)”. Manual search was carried out in selected orthodontic journals: American Journal of Orthodontics and Orthopedics, Angle Orthodontist, European Journal of Orthodontics (from 1995 to 2021.12.31). The titles and abstracts were read to find eligible studies and thus their full texts were obtained. The references in the retrieved studies were checked.

Keywords used in electronic search in databases: PubMed ((“Finite element analysis”[Mesh] OR FEM) AND (“Model”) AND (“Cleft Palate”)), Web of Science ((finite element analysis OR FEM) AND (model) AND (cleft Palate)), Cochrane Library, Google Scholar ((finite element analysis OR FEM) AND (model) AND (cleft Palate)).

## Results

### Outcome of the search process

The search identified 31 records. Screening of titles/abstracts—excluded 16 articles. If necessary, the articles were screened in more detail. 15 articles were retrieved for full texts and 11 of them were considered eligible for inclusion by 2 authors. Eventually, 11 articles were included in the qualitative analysis. Figure 1 describes the search strategy using a PRISMA flow chart.

### Material properties for structures used in the studies

The properties of the material, such as cortical bone, cancellous bone, tooth/enamel, periodontal ligament, suture, and palatal mucosa, are presented in Table 1.

**Table 1:** Material properties for structures used in studies

Study	Cortical bone	Cancellous bone	Tooth/enamel	Periodontal ligament	Suture	Palatal mucosa	Unit	Remarks
[5]	–	–	Germs 800.000	–	–	–	Pa	Materials gained
[10]	13,400	7900	19.600	–	–	–	MPa	Cortical—rest of the skull; calcellous—(whole maxilla)
[11]	13.4	–	20.2	–	0.08	–	GPa	
[12]	13.700	7900	20.000	–	10	–	MPa	
[13]	1.37 × 10^4^	7.9 × 10^3^	2.0 × 10^4^	–	–	–	N/mm^2^	
[14]	1.37 × 10^3^	7.9 × 10^3^	2.0 × 10^4^	–	0.386	–	kg/mm^2^	
[15]	1.36 × 10^4^	8.00 × 10^3^	2.07 × 10^4^	2.70 × 10^0^	1.00 × 10^0^	–	MPa	
[16]	13.426	7742	19.600	–	–	–	N/mm^2^	
[17]	1.05 × 10^4^	8 × 10^2^	2 × 10^4^	–	10	1	MPa	
[18]	12.7	–	–	–	–	–	GPa	The maxilla, simplified as the cortical bone
[19]	–	7.9 × 10^3^	2.0 × 10^4^	–	0.386	0,1	kg/mm^2^	

### Value of the Poisson ratio used in the studies

The Poisson ratio used for tissue was similar for almost all studies; for cortical, cancellous bone, and tooth/enamel it was 0.30 (except one paper), only 4 articles provided values for periodontal ligament. Suture numbers ranged from 0.30 to 0.49. The values for the palatal mucosa ranged from 0.28 to 0.45. This information is summarized in Table 2.

**Table 2:** Value of Poisson ratio used in the studies

Study	Cortical bone	Cancellous bone	Tooth/enamel	Periodontal ligament	Suture	Palatal mucosa
[5]	0.28 for all elements beside tooth germs 0.15					
[10]	0.30 for all elements					
[11]	0.30	–	0.30	–	0.49	–
[12]	0.30	0.30	0.30	–	0.49	–
[13]	0.30	0.30	0.30	–	–	–
[14]	0.30	0.30	0.30	–	0.45	–
[15]	0.30	0.30	0.30	0.45	0.40	–
[16]	0.30	0.30	0.30	–	–	–
[17]	0.30	0.30	0.30	–	0.30	0.45
[18]	0,30 for all elements					
[19]	0.30	0.30	0.30	–	0.45	0.45

### Number of nodes used in the simulation

The number of tetrahedral elements and nodes used for the creation of the model varied in different papers. For 3 articles, information about tetrahedral elements was not available. In others, the number ranged from 151.142 to 1.277.568. As concerning nodes—for 2 papers information were not available, and for the rest varied from 33.902 to 1.801.945. Information is presented in Table 3.

**Table 3:** Number of elemental nodes/tetrahedral used in the studies

Study	Tetrahedral elements	Nodes
[5]	n.a	800.000–900.000
[10]	n.a	n.a
[11]	371.605	105,357
[12]	255,140	255,270
[13]	1,277,568	1,801,945
[14]	151,142	43,142
[15]	620,950–651,995	124,784–130,988
[16]	n.a	n.a
[17]	34–105,731	49–26,719
[18]	143.084	33.902
[19]	151,142	43,142

### Clinical findings

All the information is summarized in Table 4.

**Table 4:** Clinical findings

Study	Clinical findings
[5]	Variations in the trajectory patterns in the cleft skull model compared with the normal skull on occlusal loading
[10]	Asymmetric and nonuniform stress distribution within the cleft model between the cleft and non-cleft sides due to the asymmetric skeletal maxillary defect
[11]	The transverse expansion forces from rapid palatal expansion are distributed to the 3 maxillary buttresses
[12]	Maximum displacement in the midpalatal cleft area in the BBPE, true skeletal expansion at the alveolar level without any dental tipping when compared with the conventional HYRAX expander
[13]	The protraction force alone led the craniomaxillary complex to move forward and counter clockwise, accompanied by lateral constraint in the dental arch The additional rapid maxillary expansion resulted in a more positive reaction, including both a greater sagittal displacement and an increase in the width of the dental arch
[14]	The best effect after loading maxillary protraction force, and resorption in the lower region of the grafted bone showed a better effect than resorption in the upper region of the grafted bone
[15]	It is more advantageous to perform maxillary protraction using a facemask with a miniplate anchorage than a facemask with a tooth-borne anchorage and after the alveolar bone graft rather than before the alveolar bone graft, regardless of the type of cleft
[16]	Need for customizing expansion therapy for patients with clefts depending on the patient’s age, the type of cleft present, and the desired expansion area
[17]	Visualization of bone and suture structures and explain the function and mechanism of RME on the skull with UCLP
[18]	Nilateral cleft would be expected to have an asymmetric skeletal development between the noncleft and the cleft sides as a consequence of an asymmetric functional loading pattern
[19]	RPE caused asymmetric pyramid-like displacement and deformation of UCLP; Fan-like expansion of the upper dental arch Asymmetric expansion and dispersed stress distribution on maxilla-inferior border of nasal cavity

## Discussion

The era of modern orthodontics began when Edward H. Angle published a book in 1900 entitled "Treatment of Malocclusion of the Teeth and Fractures of the Maxillae: Angle's System" creating the foundations for modern diagnostics and orthodontic treatment [20]. He is considered the father of orthodontics. Today, innovative technologies and intelligent materials have become part of the tools used by the orthodontist: digital technologies, transparent wires and brackets, aligners, robots, and computer modelling.

The driving force seems to be also Artificial Intelligence (AI) that is used in diagnostics, planning, and monitoring of treatment [21]. Digital techniques such as intraoral scanning are becoming increasingly important in orthodontic treatment. Currently, works are underway on a comprehensive digitization of the orthodontic treatment process in terms of time, cost and patient comfort. To shorten and simplify the treatment process, comprehensive practice management software (PMS) systems have been developed to plan appointments, visibility of patient information, and optimized patient communication. Digital methods allow for more innovative, patient-friendly, and time-saving treatment and are based on the direct-to-consumer business model. Although everything has recently moved in the direction of virtuality, clinical examination remains an indispensable element of therapy [22]. Therefore, it is anticipated that traditional orthodontic treatment will continue to be practiced.

Due to the use of FEM in orthodontics, any material structure (wires, brackets, rings) or maxillofacial structures (bone structures, ligaments) can be analyzed. The main assumption of FEM is the division of a larger (complex) structure into smaller sections with strictly defined physical properties (ligament, different types of bones, enamel, dentine). In this way, the response of the entire structure to the applied force is generated, e.g. orthodontic force [23].

FEM is a theoretical technique useful in the analysis of the biomechanics of maxillary protraction in UCLP patients. The maxillary hypoplasia is 3D and involves sagittal, vertical, and transverse directions with the aim of combining maxillary protraction with maxillary expansion. The comparison of the biomechanics of maxillary protraction with/without maxillary expansion was investigated. There is not much information about the biomechanical response of the maxillary complex if loaded with the protraction force of UCLP on the cleft side that was greater than that on the non-cleft side. Consequently, the existing fissure of the dental arch is enlarged (14). There are only few reports on the biomechanical effect of maxillary protraction on the craniofacial skeleton in patients with cleft and it has not been well explained clinically and experimentally using finite element methods (FEM). The basic mechanism of finite element analysis (FEA) in patients with UCLP is still unknown. There is only some information about maxillary protraction in patients with UCLP [14]. In FEA analyzes, the exceptional properties of craniofacial sutures cannot be expressed, since it was assumed that all craniofacial synchondrosis has the same histological and mechanical characteristics as the surrounding bones (19). Two methods of FEM creation are distinguished: 1) tissue-sectioning technique—generates very thin slices, destructive technique, 2) spiral CT—that enforces less scan interval, non-destructive. A skull sample for a cleft palate is usually inaccessible. The computed tomography technique was used to obtain data from a patient with UCLP (19). Because the original image of computed tomography was not enough to obtain a clear skeleton border for the generated FEM, use the window technique to obtain more readable CT images. Data from CT scans were found to be more reliable in the preparation of a digital image for FEM than from the sectioning method [19]. In the model of a cleft lip and palate, the FEA shows biomechanical characteristics of rapid maxillary expansion. It is possible to determine differences in tissue response in patients versus healthy individuals [17].

The results of some studies implied that a patient with unilateral cleft would be expected to have an asymmetric skeletal development between the non-cleft and cleft sides as a consequence of an asymmetric functional loading pattern. Pan et al. [19] investigated physiological changes and the distribution of orthopaedic force stress on the craniofacial structures of the first premolar maxillary and the crown of the first molar. Several clinical implications arise from the conducted research. It seems that asymmetric and nonuniform stress and strain distribution comparing cleft and non-clefted side [10, 18, 19, 23] and non-clefted side with higher stress and strain level [1]. The RPE procedure in UCLP patients reveals a pyramid-shaped displacement of the nasomaxillary complex along with a fan-shaped expansion of the upper dental arch [3]. The model of the patient with UCLP after ABG revealed that the best effect was obtained after loading the maxillary protraction force [14] [15]. Rapid palatal expansion forces are transmitted along three vertical buttresses [11]*.* In the craniofacial complex with UCLP, missing mid-palate and deformity of maxillary bone, the resistance to maxillary expansion mainly came from the connection between the maxilla and the pterygoid plates of the sphenoid bone [17]. However, when using FEM to assess the displacement/response of specific maxillofacial structures to an applied (orthodontic) force, many additional aspects must be taken into account. In the case of reconstruction (creation of a model) of the jaw or mandible, computed tomography is used. The computed tomography data used should have an appropriate resolution (sections of at least 0.25 mm, DICOM). In the absence of appropriate contrast and resolution, it becomes impossible to determine the boundaries of such structures: enamel, dentin, periodontal ligament. The transformation of a solid model into a model composed of a mesh of constraints and elements is the basis of FEM analysis. During the mesh refinement process, the convergence of the results with the gradual increase of bonds and elements is verified so that the voltage peaks difference between mesh refinements is 5% or less. Specialized software requires the correct representation of the mechanical properties (Young modulus and Poisson's ratio) for each mesh component [24].

Expansion therapy should be personalized according to the patient’s age, the type of cleft present (primary or secondary palate), and the desired expansion area (anterior or posterior).

The results obtained by use of FEM—are based on modeling software—therefore it is extremely important to enter all the structure data—bones, teeth, ligaments, and the applied force and boundary conditions.

### Limitations

The limitations of the present review were due to heterogeneity between the studies and a meta-analysis of the included studies could not be performed. Due to the disparate nature of the studies, only simple descriptive and stratified comparisons were reported.

## Conclusions

Despite the limitations related to the heterogeneity of the studies included in the review, it can be concluded that finite element analysis is an appropriate tool to study and predict the points of force application for better controlled expansion in patients with UCLP.

## Data Availability

All data generated or analysed during this study are included in this published article [and its supplementary information files].
